# A molecular investigative approach to an outbreak of acute hemorrhagic conjunctivitis in Egypt, October 2010

**DOI:** 10.1186/1743-422X-10-96

**Published:** 2013-03-25

**Authors:** Ehab A Ayoub, Caroline F Shafik, Anne M Gaynor, Emad W Mohareb, Magdy A Amin, Aymen S Yassin, Samir El-Refaey, Mohamed Genedy, Amr Kandeel

**Affiliations:** 1 U.S. Naval Medical Research Unit No.3, Cairo, 11517, Egypt; 2 Department of Microbiology and Immunology, Faculty of Pharmacy, Cairo University, Cairo, Egypt; 3 Ministry of Health and Population, Cairo, Egypt

**Keywords:** Egypt, Outbreak, Coxsackievirus A24 variant, Phylogenetic analysis, Acute hemorrhagic conjunctivitis

## Abstract

**Background:**

During October 2010, Egypt reported an outbreak of acute hemorrhagic conjunctivitis (AHC). A total of 1831 cases were reported from three governorates; 1703 cases in El Daqahliya, 92 cases in Port Said, and 36 in Damietta. The purpose of this study was to identify and characterize the causative agent associated with this outbreak.

**Methods:**

The U.S. Naval Medical Research Unit No.3 (NAMRU-3) was contacted by the Egyptian Ministry of Health and Population to perform diagnostic laboratory testing on eighteen conjunctival swabs from patients with conjunctivitis from El Daqahliya Governorate. Conjunctival swabs were tested by molecular methods for human adenovirus (HAdV) and enteroviruses (EV). Virus isolation was performed; the isolated virus was further characterized by molecular typing and phylogenetic analysis.

**Results:**

The majority of the samples (17/18) were positive for enterovirus and all were negative for HAdV. Molecular typing and sequencing of the isolated virus revealed the presence of coxsackievirus A24 variant. Phylogenetic analysis based on the VP1 and 3C regions demonstrated that the Egyptian viruses belonged to Genotype IV and are closely related to coxsackievirus A24 variant, reported in a similar outbreak in China in August 2010.

**Conclusions:**

This study strongly suggests that coxsackievirus A24 variant was associated with the acute hemorrhagic conjunctivitis outbreak reported in Egypt in October 2010. There is a possibility that the same strain of CV-A24v was implicated in the AHC outbreaks in both China and Egypt in 2010.

## Background

Acute hemorrhagic conjunctivitis (AHC) is a highly contagious, epidemic, eye disease characterized by eyelid swelling, tearing, and conjunctival hemorrhages. AHC is most commonly caused by two enterovirus serotypes, human enterovirus 70 (EV-70) and human coxsackievirus A24 variant (CV-A24v), and less frequently by some adenovirus serotypes [1]. The two major causes of AHC mentioned above belong to the genus *Enterovirus* of the family *Picornaviridae*[2].

Enteroviruses contain a positive sense single-stranded RNA (ssRNA) genome, 7.4 kb in length, with a 740 bp of untranslated region (UTR) at the 5^'^ end, the RNA is translated into a single polyprotein that is later cleaved into the four capsid subunits (VP1-VP4) and 2B, 2C, 3AB, and 3C viral proteins involved in RNA replication and lastly 3D the RNA-dependent RNA polymerase [2].

EV-70 belongs to the human enterovirus species D group (HEV-D) [3], was isolated for the first time in 1970 from the conjunctiva of patients with AHC in West Africa and has subsequently been detected worldwide [4]. CV-A24v is an antigenic variant of human coxsackievirus A24 strain, both classified as members of the human enterovirus species C group (HEV-C) [3], CV-A24v was isolated in 1970 for the first time during an epidemic of AHC in Singapore in which 60,000 cases were reported from September to October [5]. The outcome of viral infections caused by either EV-70 or CV-A24v is indistinguishable and both are highly contagious. Following a 24–48 hour incubation period, symptoms begin to appear as described previously and persist for three to seven days before resolving spontaneously. Although the illness is mostly nonsystemic, transient lumbar radiculomyelopathy and poliomyelitis-like illness were reported in some cases [2].

During the period 1970–1985, Southeast Asia and India reported a number of outbreaks caused by CV-A24v [6]. From 1987–1991 several large outbreaks were reported in Ghana, Taiwan, and some Central American countries [7-10]. Nearly a decade later, three major outbreaks linked to CV-A24v were reported in South Korea (2002), Malaysia (2003) and the French West Indies, Martinique and Guadeloupe (2003) [11-13]. Again from 2006–2007, CV-A24v was implicated in an outbreak of AHC in Taiwan and Yunnan, China [14,15]. During August 2010, a huge outbreak of AHC caused by CV-A24v was reported in Guangdong, China [16]. Recent studies identified the presence of four distinct genotypes of CV-A24v, designated I-IV, by phylogenetic analysis of the VP1 and 3C regions of the genome [14,16].

In October 2010, an outbreak of acute hemorrhagic conjunctivitis occurred in the Nile Delta region of Egypt, encompassing 3 governorates with 1831 suspected cases [17]. There is no published data to record any investigation of possible previous AHC outbreaks in Egypt. The objective of the present study was to identify and characterize the causative agent involved in the outbreak of AHC in Egypt, in addition identifying the relatedness of the suspected agent to similar etiologies causing AHC outbreaks worldwide.

## Results

### Virus detection and isolation results

The eighteen conjunctival swabs were tested for human adenovirus and enterovirus by rt-PCR. All of the eighteen samples were negative for human adenovirus, whereas seventeen swabs (94.4%) were positive for enterovirus RNA by (rt-RT-PCR). The seventeen positive samples were further characterized using a RT-PCR assay for the VP1 region that differentiates between CV-A24v and EV-70. All of the samples were positive for a 171 bp product corresponding to CV-A24v, and negative for a 113 bp product corresponding to EV-70. The swabs were also inoculated into cell culture to isolate the virus. MRC-5 cells were inoculated and a cytopathic effect (CPE) was observed, starting at 48 hours post infection, consisting of cell rounding and lysis. Within 4 days post infection, half of the samples (n = 9) had CPE consistent with a viral infection, at which point the virus was harvested. Bacterial growth was observed in the other nine samples which were then discarded.

### Sequencing and phylogenetic analysis results

**Figure 1:**
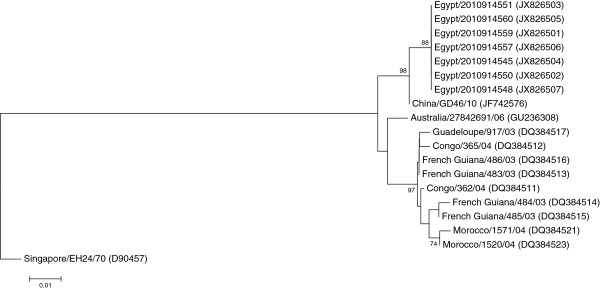
**Phylogenetic tree based on a partial sequence of the 5**´**UTR region (327 nt).** The tree was created with MEGA4 using the neighbor joining method and genetic distances were estimated using the maximum composite likelihood evolutionary model. The tree was rooted to the prototype strain of human coxsackievirus A24 variant, EH24/70 isolated from Singapore in 1970 [D90457]. Bootstrap values less than 70% were omitted. GenBank accession numbers are shown between brackets to the right of the strain name.

**Figure 2:**
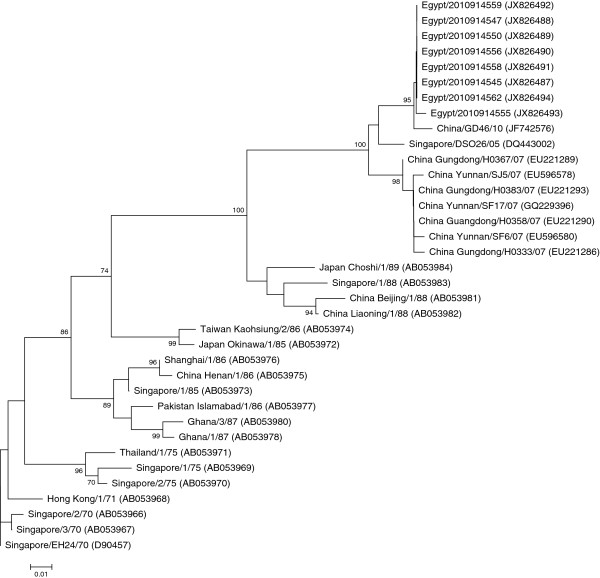
**Phylogenetic tree of the VP4 region (207 nt).** The tree was created with MEGA4 using the neighbor joining method and genetic distances were estimated using the maximum composite likelihood evolutionary model. The tree was rooted to the prototype strain of human coxsackievirus A24 variant, EH24/70 isolated from Singapore in 1970 [D90457]. Bootstrap values less than 70% were omitted. GenBank accession numbers are shown between brackets to the right of the strain name.

**Figure 3:**
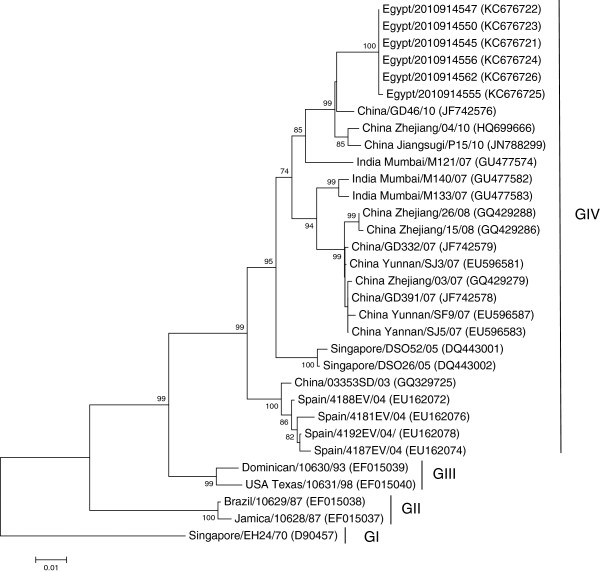
**Phylogenetic tree of the VP1 region (792 nt).** The tree was created with MEGA4 using the neighbor joining method and genetic distances were estimated using the maximum composite likelihood evolutionary model. The tree was rooted to the prototype strain of human coxsackievirus A24 variant, EH24/70 isolated from Singapore in 1970 [D90457]. Bootstrap values less than 70% were omitted. GenBank accession numbers are shown between brackets to the right of the strain name. Four Genotypes are shown on the tree.

**Figure 4:**
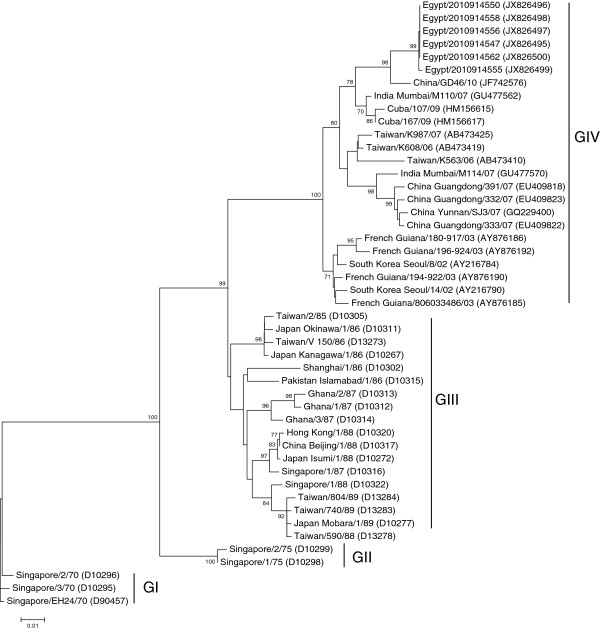
**Phylogenetic tree of the 3C region (549 nt).** The tree was created with MEGA4 using the neighbor joining method and genetic distances were estimated using the maximum composite likelihood evolutionary model. The tree was rooted to the prototype strain of human coxsackievirus A24 variant, EH24/70 isolated from Singapore in 1970 [D90457]. Bootstrap values less than 70% were omitted. GenBank accession numbers are shown between brackets to the right of the strain name. Four Genotypes are shown on the tree.

We successfully obtained sequence data for analysis from seven isolates in the 5^'^ UTR region (327 nt), six sequences were completely identical and the seventh sequence Egypt/2010914560, had 99.3% nucleotide identity to the others with no insertions or deletions. The 5^'^ UTR sequences from the Egyptian CV-A24v strains were compared to other global representative sequences from GenBank, the Egyptian sequences had a range of 87-99% nucleotide identity (Additional file 1) among the CV-A24v strains shown on the 5^'^ UTR phylogenetic tree (Figure 1). In the VP4 region we obtained sequence data from eight of the Egyptian isolates (207 nt); seven of the eight sequences had 100% nucleotide identity, the eighth sequence, Egypt/2010914555, contained a single silent mutation at nucleotide number 882 with no insertions or deletions when compared to the other identical sequences. The VP4 sequences from the Egyptian isolates shared a range of 79-99% nucleotide identity and a range of 94-100% amino acid identity (Additional files 2 and 3) among other strains shown on the VP4 phylogenetic tree (Figure 2). In the VP1 region we were able to sequence six isolates (792 nt), five of the six sequences had 100% nucleotide identity, the sixth sequence, Egypt/2010914555, exhibited a single silent mutation at nucleotide number 2586 with no insertions or deletions when compared to the other identical sequences. The Egyptian VP1 sequences shared a range of 85-98% nucleotide identity and a range of 95-100% amino acid identity (Additional files 4 and 5) among other strains shown on the VP1 phylogenetic tree (Figure 3). Analysis of the six sequences obtained from the 3C region revealed a similar outcome as discovered in the VP4 and VP1 regions with five of the sequences being 100% identical to one another, with the sixth sequence, also Egypt/2010914555, containing a single silent mutation at nucleotide number 5511 with no insertions or deletions when compared to the other identical sequences. The 3C sequences from the Egyptian isolates (549 nt) demonstrated a range of 85-98% nucleotide identity, and a range of 88.5-100% amino acid identity (Additional files 6 and 7) among other strains shown on the 3C phylogenetic tree (Figure 4). All of the nucleotide sequences from the four genomic regions shared a common ancestor, the CV-A24v strain China/GD46/10 [JF742576], an isolate from a 2010 outbreak of AHC, with 98-99% similarity based on results from BLASTn. All the VP4, VP1 and 3C sequences obtained from the Egyptian isolates had 100% amino acid identity to the China/GD46/10 strain.

**Figure 5:**
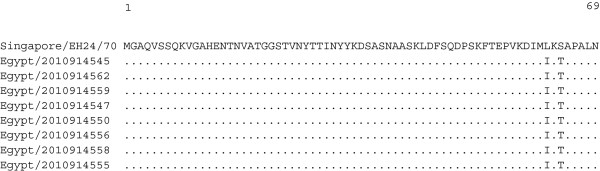
**Amino acid alignment of the VP4 region of the Egyptian CV-A24v with the prototype strain EH24/70.** Identical amino acids between strains are shown as dots.

**Figure 6:**
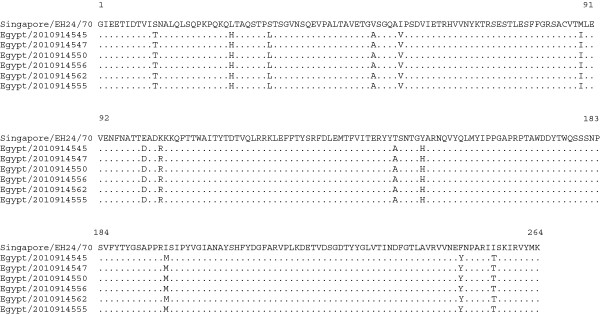
**Amino acid alignment of the VP1 region of the Egyptian CV-A24v with the prototype strain EH24/70.** Identical amino acids between strains are shown as dots.

**Figure 7:**
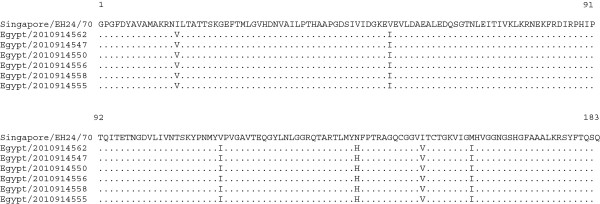
**Amino acid alignment of the 3C region of the Egyptian CV-A24v with the prototype strain EH24/70.** Identical amino acids between strains are shown as dots.

In examining the VP4 region, 34 to 35 nucleotide differences were identified between the sequence of the Egyptian isolates and the prototype strain EH24/70, with no insertions or deletions. Two of the differences were nonsynonymous substitutions resulting in a L62I and S64T amino acid change in all of the eight VP4 sequences from Egypt, while the remainders were synonymous (Figure 5). We also identified, 113–114 nucleotide differences between the Egyptian VP1 sequences and the prototype strain, with no insertions or deletions, these differences resulted in 13 nonsynonymous substitutions; S11T, L25H, S32L, V51A, I56V, M89I, E100D, K103R, T146A, Y151H, I196M, F250Y and I256T in all of the six VP1 sequences obtained (Figure 6). The 3C region of the Egyptian isolates contained 79 nucleotide differences as compared with the prototype strain, again with no insertions or deletions, the 79 nucleotide differences resulted in six nonsynonymous substitutions; I15V, V54I, V114I, N139H, I151V and M160I in all six 3C sequences obtained (Figure 7).

Analysis of the 5^'^ UTR and VP4 phylogenetic trees revealed that the Egyptian sequences are clustered into a single monophyletic group with the most closely related sequence the China/GD46/10 which was also the common ancestor based on BLASTn analysis. EH24/70 is genetically distant from all of the other isolates including the Egyptian isolates; the VP4 tree was more comprehensive than the 5’UTR tree due to the larger availability of sequences in this region from past outbreaks. The phylogenetic trees based on the VP1 and 3C regions were quite similar, the Egyptian isolates forming a single cluster with the most closely related sequence the China/GD46/10 again in agreement with the BLASTn results and the previous analysis. In these trees, the EH24/70 remained the most distantly related but the VP1 and 3C trees were more comprehensive and informative than the 5’UTR and VP4 trees, with four distinct genotypes arranged chronologically. The VP1 phylogenetic tree represented a more clearly chronological evolution of the CV-A24v than the 3C tree.

## Discussion

This study identified a viral agent associated with a small number of cases of AHC during an outbreak in Egypt. We ruled out the presence of human adenovirus and determined that a human enterovirus was present by rt-RT-PCR. We used specific primer pairs in the VP1 region of the enterovirus genome to distinguish between CV-A24v and EV-70. The amplification product consistent with CV-A24v indicated its implication in this outbreak. We further confirmed the presence of CV-A24v by sequencing four genomic regions, including the VP4, which has been cited as a suitable method for molecular serotyping in lieu of the classic homo-typic seroneutralisation assays [18], as well as the VP1 region which is considered to be the best region for phylogeny-based classification since it contains the major neutralization epitopes among the capsid proteins and its sequence was found to match with classical serotypes [19,20]. The taxonomy report obtained upon Blasting the VP4 and VP1 sequences from Egypt strongly suggested the presence of CV-A24v.

The source of introduction of this virus to Egypt is unknown, but there was media speculation that a fisherman from a city in El Daqahliya had made frequent trips to several African countries and could have imported the virus. Additionally, other media reports indicated that there was significant air pollution due to burning rice straw during the time of the outbreak which could have led to a non-infectious conjunctivitis. However, without an epidemiological investigation this was merely speculation. There were also several outbreaks of conjunctivitis in different parts of the world in 2010. Uganda and Southern Sudan reported AHC outbreaks caused by CV-A24v during June and July respectively [21]. Pakistan and Mexico reported conjunctivitis outbreaks [22,23] during September and although no etiology was demonstrated, local authorities presumed that the outbreak was of viral origin. Lastly, there was also a severe outbreak of AHC reported in Guangdong, China during the period August-October and was attributed to CV-A24v [16]. Although there is no data to indicate how the virus was introduced to Egypt, the rapid dissemination among school students (59.7% of cases were students) indicates that it probably did spread within schools once introduced.

In order to understand the relatedness of the Egyptian virus isolates, we sequenced four different genomic regions. The high degree of similarity (>99%) among the strains in each of these regions indicates that there was likely a single source or introduction that caused this outbreak. Moreover, the very high nucleotide identity (98-99%) to CV-A24v associated with AHC outbreak reported in Guangdong, China in 2010, suggests that the same virus might be implicated in both outbreaks. It is known that CV-A24v is highly contagious in a world with an incredibly mobile population.

Most studies of AHC outbreaks caused by CV-A24v used the VP1 and 3C regions as tool for molecular epidemiological analysis and four distinct genotypes of a CV-A24v strains were identified [14,16]. However, the VP1 region is more informative than the 3C region to describe the molecular epidemiology of enteroviruses, by virtue of the high recombination rates among enteroviruses and the liability of the VP1 region to mutate frequently to evade the immune response, which makes the VP1 region evolve at a faster rate when compared to the 3C region [24].

The strains shown on the VP1 phylogenetic tree (Figure 3) were grouped into four genotypes. Genotype I (G I) consisted of the prototype strain EH24/70, Genotype II (G II) consisted of strains from 1987, Genotype III (G III) contained strains from the 1990s, and Genotype IV (G IV) is composed of a more diverse group of strains isolated from 2003–2010. The Egyptian CV-A24v strains were among Genotype IV with other strains from China, India, Singapore and Spain. The 3C phylogenetic tree also demonstrated four distinct genotypes. Genotype I (G I) consisted of the prototype strain EH24/70 and other strains isolated from Singapore in 1970, Genotype II (G II) consisted of strains from 1975, Genotype III (G III) contained strains from the 1980s, and Genotype IV (G IV) is composed of a more diverse group of strains isolated from 2002–2010. The CV-A24v strains isolated from Egypt during 2010 were included in Genotype IV with other strains from China, Cuba, India, Taiwan, South Korea and French Guiana (Figure 4). The phylogenetic trees based on the VP1 and 3C regions echo the fact that four distinct genotypes of CV-A24v were previously identified [14,16].

Some sites in the affected areas are located on the Mediterranean Sea with active ports and extensive trading activity with other foreign countries, especially China; so this may be considered as a means for the introduction of this virus in Egypt. High population density and poor sanitation in some of these areas may be factors that contribute to the rapid dissemination of the virus, especially in rural locations where people may share towels and other personal belongings.

## Conclusions

This investigation strongly implicates CV-A24v strain as being associated with the conjunctivitis cases reported from Egypt in October 2010. Further characterization of CV-A24v by sequencing revealed that it belonged to Genotype IV. There is a possibility that the same strain of CV-A24v was the causative agent in the AHC outbreaks in China and Egypt in 2010. We strongly recommend increasing awareness of symptoms, treatment and prevention of AHC, as well as the implementation of control measures, including thorough investigation and response guidelines.

## Materials and methods

### The outbreak

The outbreak of AHC investigated in this study occurred in late October, and affected three adjacent coastal governorates in the Nile Delta region of Egypt. A total of 1831 conjunctivitis cases were reported by local ophthalmologists in the region, 59.7% of the cases were students. El Daqahliya Governorate, the largest of the three governorates, had the largest number of cases at 1703, of which 1042 were students. Port Said Governorate and Damietta Governorate had considerably fewer cases at 92 and 36 respectively. A viral etiology was suspected by ophthalmologists, the Egyptian Central Public Health Laboratory (CPHL) in Cairo requested the ophthalmologists to collect representative samples from symptomatic patients reported to the hospitals to identify the etiology of the outbreak. NAMRU-3 was contacted to assist in the identification of the causative agent.

### Clinical samples from AHC cases

Conjunctival swabs in viral transport medium (VTM) containing 2.5% veal infusion broth, 0.5% bovine serum albumin, 100 μg/ml gentamicin sulfate, 2 μg/ml fungizone (amphoterecine B) were collected from 18 symptomatic patients from El Daqahliya Governorate, north of Cairo. The patients’ ages ranged from 3–68 years, 15 were males and 3 were females. Reported symptoms included inflammation of the conjunctiva, increased tearing, redness and itching of the eyes. The swabs were transferred to the (CPHL) in Cairo, then to NAMRU-3 for investigation.

### Virus detection and isolation

RNA extraction was performed on 140 μl of sample using the Qiagen® QIAamp® Viral RNA Mini Kit according to the manufacturer’s specifications (Qiagen Inc.,Valencia, CA). Real-Time PCR was used to detect human adenovirus using the protocol and primers developed by the Centers for Disease Control and Prevention (CDC) Atlanta, GA, USA [25]. Real-time reverse-transcription PCR (rt-RT-PCR) was performed to detect enteroviruses using published primers [26] targeting the highly conserved 5^'^ UTR. The reaction contained 8 μl RNA, 300 nM forward primer, 900 nM reverse primer, 150 nM dual-labeled fluorogenic probe, 0.4 mM dNTPs, 4 μl buffer (Qiagen One-Step RT-PCR kit, Qiagen Inc., Valencia, CA) and 0.8 μl Qiagen One-Step enzyme mix in a total reaction volume of 20 μl. The cycling conditions were as follows: 50°C for 30 min, 95°C for 15 min, 40 cycles of 94°C for 15 sec and 60°C for 1 min and performed on an ABI7500 (Applied Biosystems, Foster City, CA). Enterovirus positive samples were then tested for CV-A24v and EV-70 using conventional RT-PCR reactions utilizing the primer pairs (S-3, AS-3) and (S-4, AS-4) targeting the VP1 region of the enterovirus genome and specific to CV-A24v and EV-70 respectively [27], the reaction contained 10 μl RNA, 1.5 mM MgSO4, 0.2 mM dNTPs, 5 μl Buffer (Promega Access RT-PCR kit, Madison, USA), 0.8 μM forward primer, 0.8 μM reverse primer, 0.5 μl Promega RT enzyme and 0.5 μl Promega DNA polymerase in a total reaction volume of 25 μl. Cycling conditions were as follows: 48°C for 45 min, 94°C for 2 min, 40 cycles of 94°C for 30 sec, 60°C for 30 sec, 68°C for 1 min followed by 68°C for 7 min. 5 μl of amplified product was visualized on a 3% agarose gel.

We also inoculated 100 μl of each of the samples onto a MRC-5 (CCL-171 American Type Culture Collection, Manassas, Virginia, USA) monolayer in culture tubes after the growth media was discarded. The tubes were incubated at 37°C in 5% CO_2_ for one hour and rocked every 15 minutes. One ml of DMEM media (Dulbecco’s Modified Eagle Medium, Life Technologies, Grand Island, NY, USA) was added to each tube and then incubated at 37°C in 5% CO_2_. The cells were observed for cytopathic effect (CPE) for seven days. When CPE was observed, the positive samples were frozen at −70°C to maintain the virus viability for further characterization. RNA was extracted from each of the isolates using the method described above, and the RNA was used as a template for the sequencing reactions.

### Sequencing and phylogenetic analysis

In order to confirm that the virus isolates were CV-A24v, further characterization and genomic analysis were performed after sequencing portions of the genome. Four genomic regions were sequenced: a portion of the 5^'^ UTR [28], the entireVP4 [18], the partial VP1 [16] and the partial 3C [29], using conventional RT-PCR. 5 μl of nucleic acid extracted from the virus isolates were used for amplification in a total volume of 25 μl containing: 1.5 mM MgSO4, 0.2 mM dNTPs, 5 μl Buffer (Promega Access RT-PCR Kit, Madison, USA), 0.8 μM forward primer, 0.8 μM reverse primer, 0.5 μl Promega RT enzyme and 0.5 μl Promega DNA polymerase. Cycling conditions were as follows: 48°C for 45 min, 94°C for 2 min, 33 cycles of 94°C for 30 sec, 52°C for 30 sec, 68°C for 1 min, followed by 68°C for 7 min. 5 μl of the amplified products were visualized on a 2% agarose, however a 1% agarose was prepared to visualize the VP1 region. The expected band sizes were 410 bp, 650 bp, 1032 bp and 674 bp for the 5^'^ UTR, VP4, VP1 and 3C respectively. Amplified products of the appropriate size were purified using ExoSAP-IT (USB, Cleveland, OH) and bi-directionally sequenced using the same primers used for amplification of the four regions cited above [16,18,28,29]. Each sequencing reaction was done using 4 μl of the BigDye Terminator v3.1 cycle sequencing kit (Applied Biosystems, Foster City, CA, USA), 4 μl of the BigDye Terminator v3.1 sequencing buffer, 2 μl of the purified amplified product, 1 μl of 3.2 μM primer in a total volume of 21 μl. Cycling conditions were as follows: 26 cycles of 96°C for 10 sec, 50°C for 5 sec and 60°C for 4 min. The cycle sequencing products were purified using the BigDye XTerminator purification kit (Applied Biosystems, Foster City, CA, USA) according to the kit instructions and then analyzed on ABI3130xl genetic analyzer. Sequences were edited using the Sequencher software V4.10.1 (Gene Codes, Ann Arbor, MI, USA). All obtained sequences were used for a database search, performed using BLASTn (National Center for Biotechnology Information, Bethesda, MD, USA) to determine which viral sequence these samples were most closely related to. Multiple sequence alignments were generated using the BioEdit software for each of the sequenced regions with some CV-A24v representative sequences from GenBank to determine the relatedness of the Egyptian isolates involved in this outbreak to other global CV-A24v isolates. Percentage similarity between sequences was calculated using the sequence identity matrix, BioEdit. The nucleotide multiple sequence alignment from each of the regions was used as an input to create phylogenetic trees with MEGA 4 software [30] using the neighbor-joining method. Genetic distances were estimated using the maximum composite likelihood evolutionary model, and bootstrap analysis was implemented with 1000 replicates. The generated phylogenetic trees were rooted to the prototype strain of human coxsackievirus A24 variant, EH24/70 isolated from Singapore in 1970 [D90457]. Bootstrap values less than 70% were omitted. GenBank accession numbers for the downloaded strains are shown on the trees.

The 5^'^UTR sequences obtained from the Egyptian isolates were given GenBank accession numbers from [JX826501-JX826507], the VP4 sequences from [JX826487-JX826494], the VP1 sequences from [KC676721-KC676726] and the 3C from [JX826495-JX826500].

## Abbreviations

ABI: Applied Biosystems; AHC: Acute hemorrhagic conjunctivitis; CV-A24v: Coxsackievirus A24 variant; CPE: Cytopathic Effect; DMEM: Dulbecco’s Modified Eagle Medium; EV: Enterovirus; HAdV: Human Adenovirus; (HEV-C): Human Enterovirus Species C; (HEV-D): Human Enterovirus Species D; NAMRU-3: US Naval Medical Research Unit No. 3; Nt: Nucleotide; rt-RT-PCR: Real-time Reverse-Transcription Polymerase Chain Reaction; rt-PCR: Real-time Polymerase Chain Reaction; RT-PCR: Reverse-Transcription Polymerase Chain Reaction; ssRNA: Single-stranded Ribonucleic Acid; UTR: Untranslated Region

## Competing interests

The authors’ declare that they have no competing interests.

## Authors’ contributions

EAA: Performed all molecular testing of samples, analyzed all data, and drafted the manuscript. CFS: Performed virus isolation and participated in drafting of the manuscript. AMG: Provided advice and analysis of results and participated in drafting the manuscript, reviewing and editing of the manuscript. EWM: Provided technical oversight throughout the study. Reviewed and edited the manuscript. MAA: Provided supervision of the work and advice in writing the manuscript. ASY: Provided supervision of the work and advice in writing the manuscript. SER: Responsible for the outbreak investigation, response and control. MG: Responsible for the outbreak investigation, response and control. AK: Team leader for managing the outbreak and manuscript revision. All authors read and approved the final manuscript.

## Authors’ disclaimer statement

The views expressed in this article are those of the author and do not necessarily reflect the official policy or position of the Department of the Navy, Department of Defense, nor the U.S. Government. Work was funded by work unit # 847705.82000.25GB.E0018. The study protocol # NAMRU3.2004.001 was approved by the Naval Medical Research Unit No. 3 Institutional Review Board in compliance with all applicable Federal regulations governing the protection of human subjects.

## Authors’ information

The main interest of the first author (EAA) in this study was to draw the world attention to the etiology of the AHC outbreak that was reported in Egypt in 2010. This is the first study to characterize the AHC etiologies in Egypt.

## Copyright assignment statement

Some of the authors are employees of the U.S. Government. This work was prepared as part of their official duties. Title 17 U.S.C. §105 provides that ‘Copyright protection under this title is not available for any work of the United States Government.’ Title 17 U.S.C. §101 defines a U.S. Government work as a work prepared by a military service member or employee of the U.S. Government as part of that person’s official duties. This is an Open Access article distributed under the terms of the Creative Commons Attribution License (http://creativecommons.org/licenses/by/2.0), which permits unrestricted use, distribution, and reproduction in any medium, provided the original work is properly cited.

## Supplementary Material

Additional file 1 **UTR identity matrix. **(XLSX 13 kb) Click here for file

Additional file 2 **VP4 nucleotide identity matrix.** (XLSX 18 kb) Click here for file

Additional file 3 **VP4 amino acid identity matrix.** (XLSX 17 kb) Click here for file

Additional file 4 **VP1 nucleotide identity matrix.** (XLSX 14 kb) Click here for file

Additional file 5 **VP1 amino acid identity matrix** (XLSX 16 kb) Click here for file

Additional file 6 **3c nucleotide identity matrix.** (XLSX 23 kb) Click here for file

Additional file 7 **3C amino acid identity matrix.** (XLSX 22 kb) Click here for file
